# Children’s Lived Experiences of Wellbeing at School in England: a Phenomenological Inquiry

**DOI:** 10.1007/s12187-023-10016-2

**Published:** 2023-03-03

**Authors:** Tania Clarke, Ruth Platt

**Affiliations:** 1 Faculty of Education, University of Cambridge, 184 Hills Rd, Cambridge, CB2 8PQ UK; 2 Anglia Ruskin University, Cambridge, UK

**Keywords:** Wellbeing, Eudaimonia, Positive psychology, Self-Determination Theory (SDT), Child mental health, School, Achievement motivation, Phenomenology, Interpretative Phenomenological Analysis (IPA)

## Abstract

This phenomenological inquiry investigated children’s wellbeing experiences at school, including their hedonic (*feeling* good) and eudaimonic (*doing* good) accounts, a distinction often overlooked. Further, while phenomenological inquiries of children’s mental ill-health exist, wellbeing, a fundamental part of mental health, is neglected. This is at odds with positive psychology which favours strengths-based approaches to studying human development. Phenomenology provides rich detail, facilitating deeper understanding of *why* and *how* certain factors affect wellbeing, as described by children themselves. A sample of 15 children (aged 9–11), attending one English primary school broadly representative of the national socio-demographic, engaged in interviews. Children’s experiences of ‘feeling good’ at school were characterised by: an interdependence on peers’ emotional states (described as ‘a domino effect’), a need to feel cared for by, and trust, adults, and desire for autonomy over their time. Children attributed mistrust in adults to adults disregarding seemingly incidental events which felt significant to children. Children experienced ‘doing well’ as equating to academic attainment, conveying a fixation with test scores, using language of ‘correctness’ and efficiency. Shame pervaded when ‘correctness’ was not achieved, with children describing being ridiculed for poor test scores. Recommendations for schools to support children’s hedonia include prioritising wellbeing curricula and emotional literacy, greater staff reflexivity, and prioritisation of pupil voice. To foster children’s eudaimonia, recommendations include the need for teachers to provide formative, personalised feedback for pupils focused on the learning process, and the need for Government to embrace a range of ways pupils can feel successful beyond academic attainment.

**Supplementary Information:**

The online version contains supplementary material available at 10.1007/s12187-023-10016-2.

For two decades, children’s wellbeing has been at the forefront of educational discourse (Ben-Arieh, 2012; Morrow & Mayall, 2009; Pollard & Lee, 2003; The Children’s Society, 2020). While wellbeing encompasses mental and physical health, the present study focuses on children’s mental wellbeing (henceforth ‘wellbeing’). To promote children’s wellbeing, schools must be acknowledged as influential contexts where children spend much formative time and, ideally, learn what it means to live well. Schools are ‘jigsaw organisms’ with a plethora of pieces fitting together, contributing to children’s wellbeing (Weare, 2006). Research suggests particularly integral pieces include children’s teacher (O’Connor et al., 2012; Zsolnai & Szabó, 2021) and peer relationships (Slee & Skrzypiec, 2016), conflict and behaviour management (González-Carrasco et al., 2019), bullying mitigation (Beynon, 2019), and whether children feel cared for (Simmons et al., 2015). Detrimental consequences of low wellbeing extend beyond children’s academic attainment (Deighton et al., 2018; Lereya et al., 2019) to longer term outcomes including health and employability (Maccagnan et al., 2019).

Despite its evident importance, the widespread promotion of wellbeing in schools has not gone unchallenged (Hayes & Ecclestone, 2009) with educators articulating a need to question its ‘taken-for-granted’ advancement (Wright, 2014). This article tackles two such taken-for-granted aspects of wellbeing promotion. First, it embraces the breadth of children’s wellbeing – including hedonia *and* eudaimonia – addressing the predominant focus on the former (children *feeling good*) and neglect of the latter (children’s experiences of *doing well)* (Huppert & So, 2011). The present article thus provides better understanding of children’s eudaimonic wellbeing experiences (Avedissian & Alayan, 2021), specifically in the school context where Government accountability measures define *doing well* as academic attainment (Department for Education, 2022). Second, it challenges the assumption that practitioners^1^* know*, and know *enough*, about what it means for children to experience wellbeing at school (Sixsmith et al., 2007). Though studies exploring wellbeing from children’s perspectives exist, they are thematic, often lacking detail of children’s lived experiences (Fattore et al., 2019).

## Theoretical Background

Three ‘waves’ of positive psychology (Lomas et al., 2021) have led researchers to conceptualise wellbeing as two distinct but overlapping experiences: *feeling* (hedonia) and *functioning* (eudaimonia) (Huppert & So, 2011; Huta & Ryan, 2010), together constituting overall wellbeing. ‘Subjective wellbeing’ (hedonia) is commonly researched according to three facets; the presence of positive emotions, absence of negative emotions, and overall life satisfaction (Diener et al., 1999). ‘Psychological wellbeing’ (eudaimonia) concerns individuals’ experiences of competence, purpose, authenticity and growth (Huta & Waterman, 2014; Ryff, 1989). Hedonia *(feeling* good) is theorised to be *a result of* eudaimonia (*functioning* well) (e.g., through engaging in personally meaningful activities or relationships) (Bauer et al., 2015; Jia et al., 2022).

Positive psychology (Seligman & Csikszentmihaly, 2000) sought to ‘rebrand’ psychology as a field, emphasising individuals’ strengths and adaptive behaviours rather than their deficits, which gave rise to the Positive Education (PE) movement in schools (Kern & Wehmeyer, 2021). Yet for individuals to *feel* good and *function well*, their core needs require supporting by their environments (Deci & Ryan, 2000; Hanley et al., 2020; Maslow, 1943; Rogers, 1979). That is, wellbeing is not only an individual concern, but a function of subject-system capacities (Nussbaum & Sen, 1993). When it comes to children, their wellbeing may be dependent on the agency (Fattore et al., 2009) or autonomy (Ryan & Weinstein, 2009) they are afforded within the systems they are embedded in, education representing one such system (Francesconi, 2018).

### Children’s Wellbeing: the Crystallisation of Key ‘Themes’

Qualitative research eliciting children’s perspectives on contributors to their wellbeing has resulted in the crystallisation of core recurring themes supported by theory. A Government-commissioned rapid review of 397 UK-specific studies exploring children’s experiences of wellbeing (Dex & Hollingworth, 2012) identified three core themes: *relationships* (feeling loved and cared for), *self and freedoms* (freedom from bullying and school-related stress, and need for autonomy), and *environment* (access to services and resources). Theoretical support for these themes includes Maslow’s (1943) hierarchy of needs individuals require to flourish, with basic needs (physiological, interpersonal and self-esteem) forming the foundation. Self-Determination Theory (SDT; Deci & Ryan, 2000), further posits individual wellbeing depends on the fulfilment of three innate psychological needs (relatedness, autonomy and competence).

International studies corroborate Dex and Hollingworth, including Sabolova et al.‘s (2020) comparative study documenting that children identify relationships and attainment as the main sources of poor wellbeing at school. Sabolova et al.’s ‘relationships’ theme included children’s distress at having no friends to play with, the need for trusting, supportive relationships with peers and teachers, and the value of shared experiences. Children also worried about having conflict with peers, being laughed at, taunted, or bullied. Elsewhere, Yetunde et al. (2014), found similar relationship sub-themes, including playing with friends and not feeling left out. Altogether, qualitative studies indicate that children as young as five situate their experience of wellbeing in relation to others; attuned to intra- and inter-individual factors (Waters et al., 2022).

Multiple studies underscore the importance of children feeling they have autonomy (Anderson & Graham, 2016; Koch, 2018; Powell et al., 2018; Simmons et al., 2015; Tuukkanen & Pekkarinen, 2022); corroborating Dex and Hollingworth’s (2012) ‘self and freedoms’ theme. At school, children emphasise their desire to learn by doing, craving active hands-on activities allowing them to explore learning topics with agency (Simmons et al., 2015). Children in Anderson and Graham’s study (2016) expressed a desire for greater control and voice, including opportunities to choose who to sit with, or be consulted about how school should be run. Similarly, children in Tuukkanen and Pekkarinen’s study (2022) prioritised being afforded opportunities for expression and self-fulfilment via hobbies, creative activities, and exploring nature. Children are especially aware of the amount of free time they are afforded at school (Yetunde et al., 2014). Conversely, children cite oppressive experiences which undermine their autonomy such as bullying, social exclusion, being overlooked or ignored by adults, being discouraged to be themselves, and being laughed at (Powell et al., 2018). Further, phenomenological research (Montreuil et al., 2020) highlights how control and sanctions are experienced by children as abusive, hindering their development of trusting relationships with adults. Children also talk about their wellbeing as being dependent on how well equipped their immediate environments are to meet their basic needs for safety, security and access to services (Simmons et al., 2015; Tuukkanen & Pekkarinen, 2022). Within their environment, children feel safest when they trust teachers to support and scaffold their learning and actively help resolve conflicts.

Few studies explicitly differentiate between children’s hedonic and eudaimonic wellbeing experiences. One exception is Vujčić et al.‘s (2019) study, whereby children perceived the quality of relationships as the main indicator of their ‘eudaimonic’ wellbeing, also underlining the degree of agency they had in their lives as important. Further, children described school achievements as influencing their longer-term wellbeing, perceiving that attainment translated into positive life outcomes. Yet some children experienced school as work-intensive and hyper-focused on attainment. Despite Vujčić et al.‘s focus on domain-general wellbeing experiences, children spoke specifically about school, suggesting schools are highly influential environments for children’s wellbeing warranting further in-depth inquiry.

### Understanding Children’s Lived Experiences: Beyond Themes

Thematic analysis studies are informative, establishing, through coding and counting, categories of wellbeing experiences (Braun & Clarke, 2014). A variety of thematic approaches exist, including classical content analysis (Leech & Onwuegbuzie, 2011), inductive and deductive analysis (Thomas, 2006). As acknowledged previously, the accumulation of thematic studies has resulted in a solid qualitative evidence base summarising aspects of children’s lives that support and hinder their wellbeing (Beynon, 2019). However, it is argued that thematic approaches may limit adults’ understanding of *how* children *experience* wellbeing (Mashford-Scott et al., 2012) in three key ways.

First, thematic analyses attribute salience to experiences coded most frequently across often large samples, with findings representing overarching perspectives. While themes have an ease and utility, they may mask important detail, particularly when it comes to complex experiences like wellbeing. Qualitative approaches such as phenomenology, alternatively examine salience through the *language individuals use* to describe their experiences (Pietkiewicz & Smith, 2014). Paying such attention to the language children use to articulate their lived experiences provides an opportunity for adults to understand their perspectives more clearly. For example, in Dunlop-Bennett et al.’s (2019, p. 111) study an eight-year-old described their experience of wellbeing as analogous to a ‘seesaw’, with the need to balance the ‘good bits’ and ‘bad bits’, while children interviewed by Curson et al. (2019) described the ‘struggle’ of homework as being ‘pushed onto your back’ (p. 36). Elsewhere, when asked to describe what quality of life means to them (Helseth & Misvær, 2010) children describe ‘positive cycles’, pinpointing friends as the most significant component of a positive ‘cycle’ compared to maladaptive cycles, described as not believing in oneself ‘at all’ (including their ability to make friends) (p. 1457). Children elaborated that maladaptive cycles led them to avoid making friends because they do not ‘dare’ to humiliate themselves.

Second, thematic coding can decontextualise children’s experiences, breaking accounts down into snippets to manage data volume and facilitate counting. Phenomenology instead analyses and presents individuals’ personal experiences in relation to wider group experiences to which they belong (referred to as ‘part-whole negotiations, elaborated later). For example, most phenomenological studies explore the wellbeing of disadvantaged groups, including young carers (Bolas et al., 2007) or those from minority ethnic backgrounds (Dunlop-Bennett et al., 2019). Others explore children’s wellbeing during specific life events such as Covid-19 (Koller et al., 2022; O’Sullivan et al., 2021), or the transition to secondary school (Curson et al., 2019).

Thirdly, phenomenological approaches focus on the *quality* of, and *meaning* individuals attribute to, their felt experiences (Koller et al., 2022), whereas thematic analysis more commonly explores individuals’ perceptions, ideas, or understanding of topics under investigation (Smith, 2003). With finer detail about *how* ‘themes’ are experienced, policymakers are better equipped to design interventions that are attuned to children’s needs. Unpacking children’s lived experiences of wellbeing (Fattore et al., 2019), their emotional texture, attributed meanings, and nuance, can provide deeper insights into themes. Phenomenological approaches facilitate this level of in-depth understanding, allowing researchers to answer questions of *why* factors affect children’s wellbeing and *how* they are experienced (Balážová & Uhrecký, 2018). Yet phenomenological inquiries with children tend to focus on mental ill-health, rather than wellbeing.

Phenomenological research investigating children’s experiences of mental ill-health (Kostenius & Öhrling, 2008; Montreuil et al., 2020; Punton et al., 2022; Woodgate et al., 2020) provide invaluable insights into what stress feels like for children; feeling ‘less than one can be’ or ‘fighting’ their bodies (Kostenius & Öhrling, 2008, p.289–290). Others uncover children’s experiences of anxiety, described as an inner turmoil leading to a ‘fractured sense of self’ (Woodgate et al., 2020, p.6). We argue such experiential detail is imperative for guiding intervention design to ensure practice captures the essence of what children need. Wellbeing is part of mental health (World Health Organisation, 2020), with longitudinal research suggesting children’s wellbeing is negatively correlated with mental *ill*-health (Lereya et al., 2022; Yoon et al., 2022). As advocated by positive psychology therefore, phenomenological inquiries into children’s mental health should include their experiences of both wellbeing and mental ill-health.

## The Present Study

This study was undertaken as part of a mixed, multi-method research project with the aim of understanding children’s experiences of wellbeing at school in England. The quantitative phase examined the relationship between pupils’ wellbeing and attainment in 21 primary and seven secondary schools. The qualitative phase involved a nested sample of two schools, the lead researcher, and county council in the design and delivery of wellbeing lessons as part of Health Education (Clarke & Hoskin, 2022). Following lessons, interviews were conducted with children eliciting their wellbeing experiences. The quantitative and qualitative phases of the project were pre-registered via the Open Science Framework (Clarke & McLellan, 2022a, b). The present article documents findings from interviews with children, to answer the qualitative research questions:

1. How do children experience wellbeing at school?
2. What does ‘doing well’ at school mean to children?

## Methodology

The phenomenological approach adopted is first detailed, followed by an overview of the participants, interview protocol and analysis approach.

### Interpretative Phenomenological Analysis (IPA)

Phenomenology – the study of how individuals make sense of their world and the meaning they attribute to their experiences – informed this study’s design, data collection and analysis. The researchers sought to understand the meaning children ascribed to their wellbeing experiences using a ‘person-centred’ approach (Brundrette & Rhodes, 2014). First-hand experiential accounts derived directly from children are essential in research conducted with the aim of understanding children’s wellbeing (Dunlop-Bennett et al., 2019), countering the ‘adultcentric’ methods often used (Ben-Arieh, 2005; Sixsmith et al., 2007).

IPA (Smith, 1996) was chosen given its widespread use in psychological health research (Larkin & Thompson, 2011) exploring adults’ (Chauhan et al., 2020) and children’s mental ill-health experiences (Eide et al., 2020; Stoll & McLeod, 2020; Wadman et al., 2018). Committed to three core ways of understanding (phenomenology, hermeneutics, and ideography) IPA pays attention to the *language individuals use* to assign meaning to their experiences (Pietkiewicz & Smith, 2014). IPA theorists argue that one cannot understand the meaning of language without an understanding of the speaker and the context in which they are situated (Schleiermacher, 1998; Smith, 2007). The present study involved the lead researcher being embedded within the school for two weeks prior to data collection. The pre-interview period involved the researcher engaging children in wellbeing lessons to understand their school experiences (Clarke & Hoskin, 2022). The researcher thus familiarised their self with children’s unique school context through prolonged engagement as part of the research project, lasting approximately one year.

#### Putting the ‘I’ in IPA

IPA embraces the unique contribution made by researchers to the meaning-making process and subjectivity involved in interpretation (Smith, 1996). IPA researchers should actively acknowledge and ‘bracket’ fore-conceptions, ensuring their attention is devoted to participants’ meaning first. Accordingly, the researchers interrogated how their pre-conceptions, theoretical orientations, and impetus might shape data interpretation (Brocki & Wearden, 2006; Gadamer, 1981). The lead researcher, a psychologist of education with assessment and curriculum development experience, conducts research on children’s wellbeing at school. The second researcher, an experienced Primary school educator and school leader, is responsible for teacher training in higher education. Both researchers’ backgrounds pre-disposed them to particular ‘readings’ of children’s accounts. The authors remained alert to how their pre-conceived ideas might influence their interpretations. For example, the researchers understood wellbeing as constituting hedonia and eudaimonia, and were alert to potentially ‘adultcentric’ interpretations of each throughout analysis.

#### Analytic Framework: Feeling Good and Doing Well

While the researchers remained open and attentive to whether pre-conceived conceptualisations of wellbeing resonated with children’s accounts, the guiding analytic framework that informed the interview schedule^2^ design distinguished between children’s experiences of *feeling* (hedonia) and *functioning* (eudaimonia) (Huppert & So, 2011). Research establishes the relevance of these two wellbeing branches for children (Gentzler et al., 2021). It further shed light on the extent to which the school system *affords* opportunities for children to feel they are *functioning* well and as a consequence, *feel* good. Accordingly, interview questions elicited children’s hedonic and eudaimonic experiences (‘things at school’ that made children ‘feel good’/’less good’ and ‘feel they were doing well’/’less well’, respectively). Focusing children’s accounts on ‘things’ at school that facilitate their wellbeing enabled research question 1 (pertaining to *how* children experience wellbeing) and research question 2 (*what* it means for children to do well), to be answered.

#### Exploring Shared Context: Group IPA

Group interviews enable researchers to derive rich multi-perspective accounts, serving the aim of the present study to understand the plurality of children’s wellbeing experiences. Traditionally, IPA has been idiographic in focus. Yet psychologists highlight IPA’s value in capturing shared experiential reflections of naturally-occurring groups (Tomkins & Eatough, 2010), suggesting group interactions can serve as catalysts for sharing collective memories and opinions, eliciting responses participants may not have otherwise shared. Palmer et al. (2010) claim group interviews unearth rich experiential insights not afforded in individual interviews as group dynamics encourage the sharing of mutual experiences and co-constructed understandings. It was assumed that children were capable of such co-construction through discussion facilitated by the researcher.

Children’s wellbeing is culturally contingent (Fattore et al., 2019) with national-, local-, and school-level contexts being highly influential. Sensitivity to context is critical for IPA, regardless of whether interviews are group or individual (Shinebourne, 2011). Aside from those reviewed by Dex and Hollingworth (2012), few English studies exist, with fewer conducted within schools phenomenologically. To impact educational practice, local case studies grounded in English school contexts are required. Schools being distinct ecological systems (Bronfenbrenner, 1979) shared by children, and the known influence of school climate on children’s wellbeing (Langford et al., 2017) made group interviews critical for understanding the experiences of children within the same class and school.

### Participants and Procedure

Children across schools were expected to report overlapping but different experiences, therefore schools were treated as case studies. This article documents findings from five interviews with children attending one school (Silver Birch)^3^, situated in a county identified as a social mobility ‘cold spot’, performing within the worst 20% of counties in England (Social Mobility & Child Poverty Commission, 2016). Table 1 summarises the school’s socio-demographic composition. In total, 15 children who volunteered (aged 9–11, *M* = 9.9; Table 1) were chosen to participate. Children were invited to create their own pseudonyms. The lead researcher prioritised ensuring the sample was as varied as possible in terms of demographic backgrounds (including a range of pupils of different ethnicities, eligibility/ineligibility for FSM, with/without SEN/EHCPs). Additionally, children chosen included a mix of pupils who were vocal/less vocal in wellbeing lessons, providing quieter pupils with opportunities to contribute in a smaller group setting. Nonetheless, particular groups (e.g., pupils with SEN/EHCPs) are not represented in the final sample, owing either to these pupils not volunteering to participate in the interview phase of the research, or an absence of these pupil groups at the class cohort-level (e.g., pupils from certain ethnic backgrounds). Findings should be interpreted with this in mind, given the present sample is missing accounts from particular disadvantaged groups (see Limitations).

**Table 1:** Pupil demographics at Silver Birch compared to national averages

Level	School	% pupils eligible for FSM	% pupils with a SEN or Education, Health and Care Plan	% pupils requiring SEN support	% pupils with EAL
Primary	Silver Birch	Below average	Average	Average	Above average

Free School Meals (FSM); Special Educational Needs (SEN); English as an Additional Language (EAL). Information derived from publicly available Government database

**Table 2:** Participant information

Name	Gender	Age	EAL?	SEN/EHPC?	FSM?	Ethnicity	Interview group
Mayo	Female	9	N	N	N	Any other mixed background	Group 1: Year 5 Girls
Gina	Female	9	N	N	N	White British	
Fion	Female	9	Y	N	N	Any other White background	
Will	Male	9	Y	N	N	Any other White background	Group 2: Year 5 Boys
Eric	Male	9	N	N	N	White British	
Alex	Male	9	N	N	N	White British	
Bob	Male	9	N	N	N	Any other White background	Group 3: Year 5 Boys
John	Male	9	N	N	N	White British	
Robert	Male	9	Y	N	N	Any other White background	
Shannon	Female	10	N	N	N	White British	Group 4: Year 6 Girls
Maisie	Female	10	N	N	N	Any other White background	
Kelsey	Female	10	N	N	N	White British	
Asher	Male	10	N	N	Y	Any other Black background	Group 5: Year 6 Boys
Oman	Male	10	N	N	N	Bangladeshi	
Luke	Male	11	N	N	N	White British	

Pseudonyms used for pupil names. Free School Meals (FSM); Special Educational Needs (SEN); Education, Health, and Care Plan (EHCP); English as an Additional Language (EAL). Information derived from school administrative data

Semi-structured interviews conducted by the lead researcher followed a six-part schedule^4^: (1) *Introduction*, reminding children of research aims, their right to withdraw and safeguarding procedures, (2) *A warm-up exercise* reminding children of the range of emotions they might experience at school, (3) *Children’s experiences of ‘feeling good’ and ‘less good’ at school*, (4) *Children’s experiences of ‘doing well’ and ‘less well’*, (5) *Children’s experiences of learning goals*^5^, and 5) *Closing thoughts* (opportunity for children to ask questions and sign-posting to mental health services).^6^ On average, interviews lasted 34-minutes.

Feelings individuals attribute to their experiences are key sources of meaning in phenomenology (Smith & Osborn, 2015). Pre-prepared follow-up questions funnelled children’s responses to better understand their feelings. Phenomenological inquiries additionally often use visual aids such as feelings grids to help children articulate their feelings (Koller et al., 2022). A warm-up exercise using post-it notes therefore supported children to identify and organise feelings into ‘good’ and ‘less good’ feelings they might experience in school prior to any interview questions. Children were familiar with a range of ‘good’ and ‘less good’ feelings having previously discussed them as part of pre-interview wellbeing lessons (Clarke & Hoskin, 2022) whereby a feelings grid was used. During the interview, children often referred to their post-it notes when asked follow-up questions like ‘how did you feel?’.

### Ethical Considerations

Schools interested in taking part in the qualitative phase at initial recruitment (email to headteachers) were invited to participate following the quantitative phase. Five schools expressed initial interest, resulting in two schools due lack of follow-up response or unavailability. At the project’s culmination, all schools were provided with research reports, lesson plans, and a teacher training webinar. Parental written consent and pupil assent were obtained. Pupils were invited to participate in interviews following wellbeing lessons. The researcher verbally communicated the interview aims, how their responses would be used and assured their anonymity. Three-to-one interview groups were organised by teachers using their knowledge of social dynamics most conducive to all pupils feeling comfortable speaking. Children were interviewed alongside same-gender peers to harness shared experiences and ensure they spoke openly (Adler et al., 2019). Interviews were audio recorded solely for later analysis, available only to researchers involved in the study. Recordings were stored on an encrypted drive on a password-protected computer. Transcripts were anonymised.

### Analysis Protocol

**Fig. 1:**
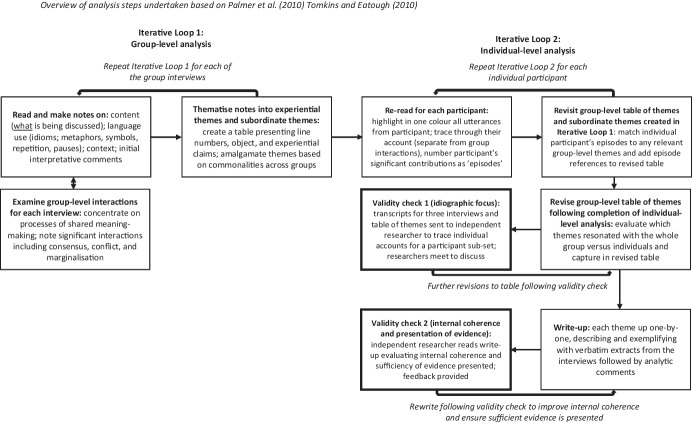
Overview of analysis steps undertaken based on Palmer et al. (2010) Tomkins and Eatough (2010)

An analysis protocol created prior to analysis was followed (Fig. 1) adhering to IPA guidelines (Pietkiewicz & Smith, 2014) expanded to accommodate the eight steps for group IPA proposed by Palmer et al. (2010). Iterative loop one involved reading and note-making (for content, language, and initial interpretative comments) at the group-level forming initial Group Experiential Themes (GETs) for each interview. Iterative loop two involved the researcher re-listening to interviews for each child, assessing the salience of GETs for each of their significant ‘episodes’ and creating tables for each child’s Personal Experiential Themes (PETs).

#### Part-Whole Negotiations

**Fig. 2:**
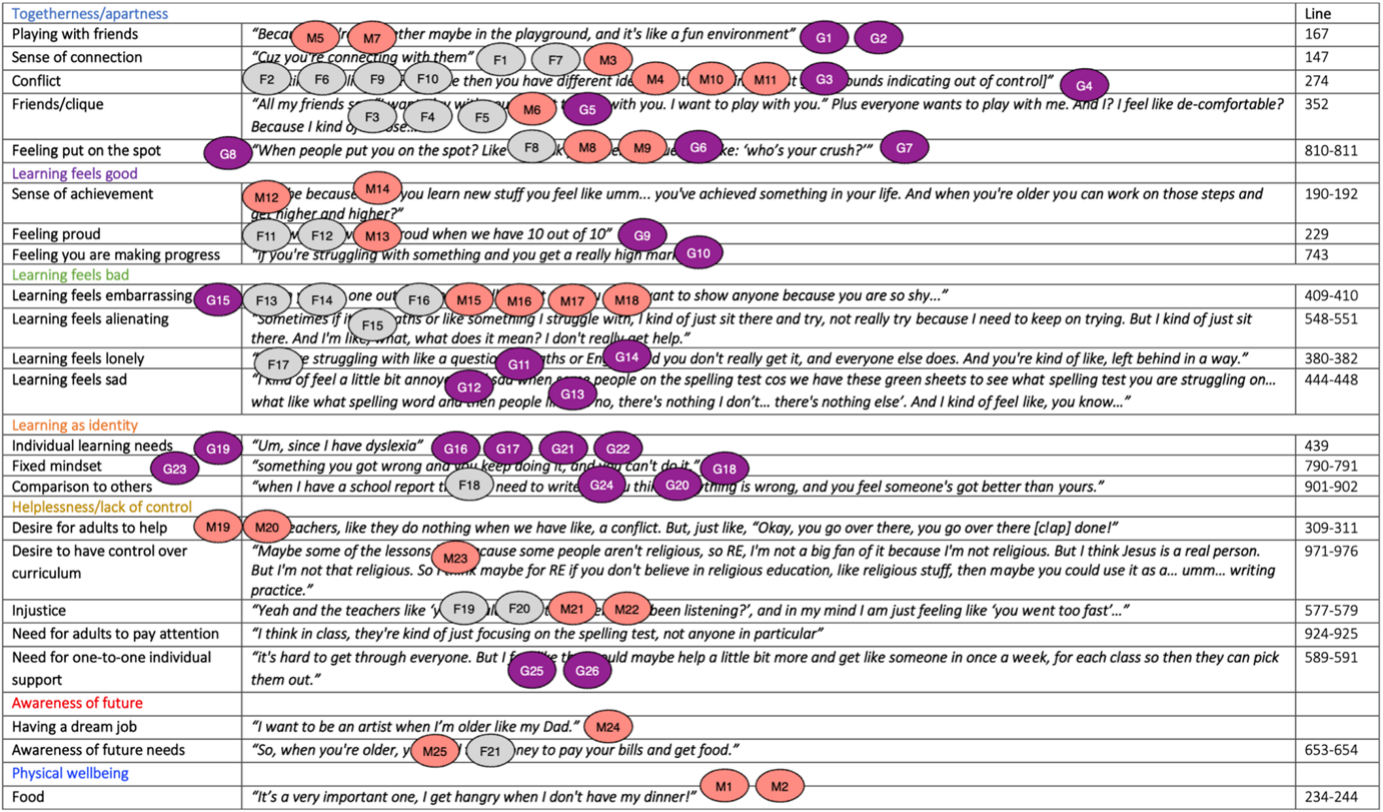
Mapping of Personal Experiential Themes (PETs) onto Group Experiential Themes (GETs) – conducted for each group interview. *Note*: GETs with overlayed PETs for interview group one. Each circle represents one ‘episode’ spoken by one child in the interview, e.g., G1 is Gina, episode 1. The three different colours represent each of the three different children’s episodes. For example, the purple circle ‘G1’ represents one of Gina’s ‘episodes’ where she talked about ‘Playing with friends’

IPA requires researchers to attend to both the ‘part’ and the ‘whole’ as articulated in Husserl’s (1999) ‘hermeneutic circle’. Different part-whole relations were attended to during analysis, the most pertinent being each individual child and their part within their interview. During the first iterative analysis loop, the researcher interrogated the ‘groupness’ of GETs to disentangle individual- versus group-level claims, attending to moments of conflict, complimenting, comparison and pronoun use (Phillips et al., 2016). Iterative loop two involved mapping each child’s PETs onto the GETs for their group (Fig. 2). Mapping facilitated visual representations of children’s experiences across the interview groups. For example, ‘Learning as identity’ was a superordinate theme unique to Gina’s wellbeing experiences at school, with Fion sharing one episode under the sub-ordinate theme ‘Comparison to others’ (Fig. 2). After mapping, a revised GETs table was produced for each interview group. After completing all steps for each interview group, final GETs were compared and amalgamated into a master GETs table including superordinate, subordinate, and child-specific themes. In the present article, superordinate GETs are presented, with any differences in individual children’s’ experiences of them noted.

#### Validity

The validity of findings from IPA studies can be upheld in three ways (Shinebourne, 2011): sensitivity to context, transparency (clearly describing all stages of the research) and conducting mini- or independent audits (independent researchers’ scrutinising analyses). In this study, sensitivity to context was attended to by analysing children’s verbatim accounts within their specific school context (case study approach). Transparency was upheld by pre-registering the study via the OFS, including clear documentation of each study stage. Finally, two mini-audits, one during and another at the culmination of analysis was conducted by two independent researchers. The mini-audit during analyses involved the second author evaluating whether initial themes were justified by the data (Osborn & Smith, 1998). The second audit involved a third researcher checking the final write-up for internal coherence and presentation of evidence (Smith, 1996).

## Findings

### Feeling Good

Amalgamation of final GETs across interview groups resulted in four superordinate GETs summarising what made children ‘feel good’ at school (research question 1; their hedonic wellbeing): together-apart, ‘a pen can be stronger than a sword’, ‘it feels like they don’t care’, and ‘they could maybe help a little bit more’.

#### Together-Apart

Children described experiences of being together and apart as making them ‘feel good’/’less good’, including their happiness being dependent on friends’ happiness, “connecting”/playing with friends, inclusion/exclusion, making “new” friends, and school being their central source of friendship.

Forming new friendships and feeling included were important experiences for Will and Eric:

Will: If I make a new friend?

Eric: If I get invited to play with someone?

For Alex, having “a person in your life” was described as an essential “need” to be “happy”.

Alex edits his own meaning, first describing “a person” and then “a friend”, suggesting that it is not just any “person” he needs to feel “happy”.

Alex: You need a person in your life, a friend in your life, otherwise you’ll just be…

Will: A zombie, you’re gonna be a zombie

Alex: You’ll just not be that happy because you have no one to talk to.

Will: Cuz I think you could be home-schooled. But then you wouldn’t be able to meet people…

Alex: Like I had… in London where I used to live. My neighbour… I think his name was Turner, I can’t really remember. But he was home-schooled by his mum. His mum was teaching him. And since he was being home-schooled, he didn’t really know how to, I don’t know, interact with people, honestly, because he didn’t have exact friends, so he was sort of mean to me a lot of the time.

Alex’s meaning is further revealed when he elaborates, talking about his old neighbour – seemingly fitting Will’s description of “a zombie” – who did not have “*exact* friends”. Alex attributes Turner’s ‘meanness’ to him not being socialised at school “so he was sort of mean”, suggesting school is a place where children learn how to treat others well. The centrality of friendships for feeling ‘good’ at school was also articulated by Asher and Robert. Asher attributed the importance of school for him to being an only child.

Eating and playing with my friends. Especially eating… Playing with my friends because not only is it the fact that I haven’t seen them over lockdown term and things for days and the fact that I just enjoy playing with my friends since I have no siblings.

Robert’s experience echoed Asher’s, commenting that “if there wasn’t any school, I bet I wouldn’t have friends. Yeah, they would have some but…”. Notably, Robert’s follow-up suggests school presents an abundance of friendship opportunities compared to home (where he may only have “some”). Others, like Maisie, Asher, and Luke, alluded to a kind of emotional interdependence whereby their own wellbeing was dependent on others feeling good.

I was also gonna say, um, a lot of people talk about like, a domino effect? Like, where you make someone happy and then it also makes you feel good… if you’re hanging out around your friends a lot, like they’re mainly around you in the day, and they’re happy, like they could be happy for another reason. But if they’re mainly around you, and they’ve actually stayed happy, that shows that like, they actually enjoy being around you.

(Maisie)

Asher: And the other thing was sometimes when my like… when one of my best friends feel lonely or sad, it kinda makes me feel sad… or bad

Luke: That’s also with happy, when your friend’s happy it makes you feel happy

Asher elaborated on this experience using an analogy of each child having their own ‘bucket’ inspired by a book^7^ pupils read in class:

Asher: Yeah it’s like when you fill up someone else’s bucket, your own bucket gets filled. Do you read? Do you know the story…? So basically, imagine everyone’s carrying around an invisible bucket, and what happens is... when you’re happy, your bucket’s full and when your bucket’s empty you’re sad and then there’s some people that try to take your happiness and put it into their bucket but it doesn’t work, so everyone feels sad and sometimes when you fill someone else’s bucket your bucket also is filled.

Oman: So from that it’s basically if you make other people happy, you’re going to probably feel happy yourself. And if you do make other people feel sad, you’re not necessarily going to be feeling happy… it was a book… that was read to us.

Children’s experiences of togetherness contrasted with the uncomfortable, “sad” feelings associated with separation from friends, including being excluded from play, or “split” apart due to conflict.

Well I was going to say not having anyone to play with… Makes me feel like left out, and sad.

(Kelsey)

Fion, Mayo and Gina described feeling torn, having to “go between” friends, unable to “go on anyone’s side”. Fion’s verb “split apart” describes a rupture between friends caused by their “different ideas” which spirals out of control and is overwhelming, as she enacts (“wooooaaah”).

Fion: Um, when you have conflict with your friends.

Researcher: So what happens?

Mayo: You kind of split apart? And like then you have different ideas and then it kind of just goes… wooooaaah!

Fion: You get angry and you get sad.

Mayo: You get depressed.

Gina: Or like if two friends out of three, like fall out, and then one has to kind of go between… two

Mayo: Yeah, that’s happened towards me before.

Fion: Yeah.

Gina: Yeah, cuz you don’t want to go on anyone’s side but then you don’t wanna be mean to them.

#### A Pen Can Be Stronger Than a Sword

Children shared a range of experiences of being victims of cruelty from “being bullied” to peers talking “about you behind your back”, “passing notes”, saying “mean things” or feeling humiliated when they get a “question wrong” and “the whole school knows”. Alex, Will, and Eric co-constructed their understanding of others’ cruel at school in an exchange between them initiated by Alex and catalysed by Will’s use of the metaphor “a pen can be stronger than a sword”. Eric agrees with Will, discerning the hurt he experiences “by feelings” to physical hurt (“like falling”). Will elaborated, describing the lasting power others’ words have on him, unable to get them out of his mind.

Alex: I wouldn’t feel good if someone was mean to me.

Researcher: Could you tell me a bit more about when people are mean to you?

Will: They could say…

Alex: They could just like say mean things.

Will: Just like cuz… have you heard the expression ‘a pen can be stronger than a sword’? It means… it means words can be stronger than violence.

Alex: It’s true.

Eric: It is true.

Researcher: Tell me about that. So, people using nasty words?

Will: So, like, ‘you’re really bad at this’, ‘you’re stupid’, or…

Eric: I sometimes, like, get more hurt by feelings than like, actually like falling or anything.

Researcher: So… we can have like a physical injury, but we can also *feel* hurt.

Alex: A mental injury. Well not really ‘mental’, but...

Will: Like, in your mind… Cause sometimes when somebody does it you just keep on thinking about it and thinking about it. But even if you try not to, you’re like, I don’t want to think about it, it’s bad! You just, keep on thinking about it.

Alex: Or it’s just like someone, you’re really good at this thing, and then someone says you’re not and then you get the answer wrong or something. Because they said you’re bad at it.

Will: And you actually kind of believe them?

Will’s description of his mind cycling through “mean things” people said as he rhythmically repeats “thinking about it and thinking about it” and “you just keep on thinking about it” conveys an intrusiveness. Will describes the thoughts as unwelcome (“I don’t want to think about it”) but despite trying “not to”, he cannot control his mind rehearsing the “bad” words others said to him. Alex also describes internalising the “mean things”, but there is a difference in the impact words have on him compared to Will. The impact Alex describes is self-doubt with others’ words (“you’re really good at this thing, and then someone says you’re not”) causing him to “get the answer wrong… because they said you’re bad at it”. Like Will, the words have a powerful hold over Alex’s mind. Eric’s short, affirming turns throughout this exchange suggest he resonates with Alex and Will’s experiences, but his own unique experience is articulated last.

Um… this isn’t actually at school. But since I have divorced parents, when my mum sometimes says stuff that’s sort of blaming my dad, I don’t really like that.

For Eric, his home circumstance might mean he is more susceptible to being “hurt by feelings” (his words). Asher’s experience of cruelty was most worrying, describing historically “being bullied… for a couple years of school” when he “then told *[his]* mum and she sorted it out”. Though Oman and Luke also shared experiences of being “mentally” “hurt”.

Oman: When we’re hurt?

Researcher: When you’re hurt? Like physically?

Oman: Um yeah, and mentally…

Asher: That one time when –

Researcher: Tell me about what it means to feel mentally hurt.

Oman: Yeah like things like erm… if they talk about you behind your back, like erm… like maybe bad things, and you don’t know what they’re saying and they act, and they behave like strangely around you.

Luke: Yeah, like when they like pass notes. It’s not like really a thing in this school. But I’ve seen it in others

Oman: Like passing notes like, that’s the same thing. They’re talking about you badly, but like, but they’re not telling you what they’ve said

Asher: Talking about you behind your back

Researcher: How does that make you feel?

Luke: Upset

Oman: Yeah

Luke: And lonely

More insidious experiences of cruelty were co-constructed by Bob, Robert, and John, who described feeling “embarrassed” when “people make fun” of them because they “don’t understand *[their]* Maths”.

Bob: So like if you don’t understand about your Maths, it kind of makes you feel like you don’t know a lot of stuff… and sometimes you get embarrassed by that or people make fun of you because of that… Sometimes it’s not really making fun of it’s more like boasting like ‘oh, I’m really good at this’”

Robert: Like once, I was with my friend, we were working and… there was a times table like, it was like two times eight and I forgot it and he said it, and… that basically made me feel a bit sad that like I forgot the basics.

John: Oh yes yes so like I think I forgot yeah I got something I got something two times eight wrong and like the whole class knew about it and it was a little bit…

Bob: If it happened to me, I’d probably be mad at everyone.

John: Well, it depends. Like, like, if you got like, just one question wrong, or just like…

Robert: Maybe not embarrassed? Because not everyone knows.

Robert’s experience of being humiliated by his “friend” was both similar and different to Bob’s, indicated by Robert’s questioning of Bob feeling “mad at everyone” for humiliating him. John then validates Bob’s experience helping explain why Bob’s anger is justified because “sometimes” they confide in their friends about getting the answer wrong and then “they tell their friend”, which John repeats three times, portraying the insidious spreading of his score before being cut-off by Bob who abruptly finishes their shared story “and then the whole school knows”.

John: Yeah. But then sometimes this happens, like when you tell when you tell your friends what happened. And they tell their friend even though they’re not your friends. And then they tell their friend, and they tell their friend and then they tell their friend… And then it works around the class and the school.

Bob: And then the whole school knows

#### It Feels Like They Don’t Care

Some children expressed feeling that adults at school did not care about them, describing different experiences that had led them to believe this in contrast to the kindness of friends who showed they cared through words of appreciation:

Maybe when your friends are nice to you, or you’re nice to your friends? …when your friends say like, thanks, or you’re welcome.

(Bob)

A friend says thank you, because you did something and they say thank you and say: “You’ve been a real friend… you’re a good friend”.

(Will)

For Will, his experience stood in opposition to his felt lack of care by adults at school. Specifically, Will felt “sad” and “angry” when support staff did not “care” about him being “physically hurt”, suggesting seemingly incidental moments in the playground feel big to children.

John: Oh, yeah. Like… when I get physically hurt. And then, like, the TA is like, at lunchtime yesterday, I got run into this person… we were playing then we just got… I don’t know. And then because it was Timmy and he has special needs… and then he got me into a head lock and ran me into the ground, and then they trampled on me. And the TA just told me to get up and I’ll be fine.

Researcher: How did it make you feel?

John: Sad, angry.

John’s vivid and brutal description of being “trampled on” by Timmy suggests he felt insignificant and helpless. He describes feeling disregarded by the Teaching Assistant (TA) who “just” told him to “get up”, implying he needed more care from them:

“Maybe the TAs – like Miss Candy and Miss Cane – will like help you when you get hurt… just like just don’t say get up… whenever I get hurt… just like ‘get up, you will be fine’ and stuff… It feels like they don’t care about me? And like I sometimes think oh, yeah, like my arm… What are they gonna say? …If I actually break my arm, like, what would they do? …But It’s not the teachers. It’s the TAs, it’s like, like, it’s like at green time^8^ and break time.”

John expressed outrage at the TAs’ apathetic response to him being “trampled” through his rhetorical questioning “what would they do?” if he “broke” his arm. John feels overlooked, perhaps even unsafe, due to their negligence and lack of care, questioning the extent to which the TAs “care about” him. Notably, he clarifies it is the support staff, “not the teachers” who he needs more “help” and “care” from.

Access to physical resources and feeling considered at a broader school level was another permeation of children’s need to feel adults care about them.

Shannon: …this isn’t just about feeling better at work, but just like happy in general… I’d like a climbing frame or something like that in the playground since Key Stage One have got one, and they’ve even got new ones but we just have like gravel…. *[something]* that isn’t the basketball because that’s like always taken up… so you can’t always play.

Maisie: …not necessarily a climbing frame, because we don’t actually have space for that in our playground. But it’s kind of unfair because they completely redid the Key Stage One playground and, and we got nothing.

Shannon and Maisie experience a scarcity of resources illustrated through their co-constructed description of the playground: a small “space” with “just” “gravel” and a “basketball” that is “always taken up” contrasted with their younger peers who are “completely” taken care of with their “new” playground. The children’s contrasting seemingly expresses incredulity and desire for adults to consider everyone’s needs equally.

#### They Could Maybe Help a Little Bit More

Feeling helpless or frustrated characterised many children’s experiences of school, with many longing for individual support and feedback on their schoolwork from teachers:

“And about my dyslexia. I mean, it is hard because I know loads of people in the school who have it, and it’s hard to get through everyone. But I feel like they could maybe help a little bit more… maybe just get someone in to help people with dyslexia kind of learn a bit more? …in class, they’re just focusing on the spelling test, not anyone in particular… they could get someone once a week for each class. And they just help you.”

(Gina)

“It happened in maths yesterday, there was a question and… I didn’t understand it. And I asked the teachers, I put my hand up… and she was working on with someone. And she said ‘wait a minute’, and she didn’t… like just ignore me… but I would like that she comes immediately, but I feel she doesn’t because she’s like helping someone else.”

(Robert)

“…when they tell you like in your book that you need to work on it, I feel like they should do that and help you work with it a bit more. Other than like, because we normally go on to the next lesson after they, like, quite often... So maybe if we spent a longer time, we would master it a lot easier, and we’d bring it into the future with us.”

(Luke)

Mayo’s experience of helplessness after ‘failing’ “a test” was particularly strong, blaming her teachers and feeling let down. Once again highlighting children’s incredulity at their perceived lack of adult support:

“…the annoying thing is when… If the teacher says you failed on a test… Sometimes you’re just like, you really want to say something. And most of the times in my brain when the teacher says that I, I really want to say to her, ‘you’ve failed… teaching me’. If you fail on a test, that means a teacher failed teaching you”

Mayo attributes her ‘failure’ to her teachers’ “teaching”, holding them accountable. Her ‘annoyance’ at the teacher, expressed through her retaliatory redirecting of the teachers’ words (“failed”) back on them (“you’ve failed teaching me”). Mayo’s experience, seemingly charged with frustration and a defensiveness, is perhaps due to feeling judged by her academic performance, or perhaps the adult-child power dynamics meaning she cannot challenge her teacher directly.

For Mayo, a lack of adult support was not limited to schoolwork, expressing feeling helpless, unable manage conflicts alone, and unsupported by teachers.

Mayo: You could ask the teacher for help?

Researcher: Have you done that before?

Gina: I think we’ve asked the teacher then for like…

Mayo: Only one time… they… the teachers, like they do nothing when we have like, a conflict. But, just like, “Okay, you go over there, you go over there [*clap*] done!”

Shannon felt helpless when their breaktimes were “cut” short unfairly because of their peers’ behaviour, as did Kelsey:

Shannon: …our class has a tendency to chat… so we didn’t have our full break because people were talking so we couldn’t go out… I find it very frustrating because it’s cutting into everyone’s play time… it would be more fair if the people who have been naughty or holding people back they had to stay till they’d quietened or whatever, and the other people go, because then the people who are behaving wouldn’t have to wait for the people who weren’t behaving.

Kelsey: I think it’s all just reaaally boring… being sat on your bum waiting for everybody to stop chatting!

Shannon’s resentment towards her peers is expressed via the verb “cutting into” which conveys a harshness. Further, Shannon, Kelsey, and Masie observe how other pupils are comparatively treated:

Shannon: …also a thing that can be annoying, I don’t know if it’s normal for you two, but you might have noticed that class three get playtime in the afternoon, and they didn’t do that when we were in class three… we don’t get a break from anything.

Kelsey: This was just this last week, but I found it really quite unfair that the Year fives actually got their break time whereas the Year sixes didn’t get break time because we had bikeability in the morning-

Shannon: Oh yeah, because if you have bikeability in the morning then you don’t get-

Kelsey: Any break time-

Maisie: And you don’t get a snack either, so you’re super hungry. Because we both had green time and we both had bikeability so that’s equal. And then the Year fives got break time and we didn’t and then they also got to eat their snacks and stuff.

Shannon: So cause, you don’t even get… you don’t get to stop and just have a pause… have a snack and have a chat or something with your friends.

All three express a lack of agency in being ‘given’ a rest or not (“we don’t get a break”, “Year fives… got their break) and the adult-child power differentials which Kelsey feels are “really quite unfair”. The children evaluate how “equal” adults’ treatment of them is, feeling helpless at not being given what feel they deserve even when, they are “super” hungry. They do not describe adults communicating *why* their rights are being given/withheld. Comparatively, children enjoyed feeling they had autonomy, such as ‘getting to’ “interact” and do “something hands on” in lessons:

“…being active… I feel like if you don’t really get to interact with the lesson, you’re just sitting there and listening to the teacher go on and on. But if you actually get to do like something hands on, you just have more fun… *[in]* science and art we really get to… in art we get like mannequins that we can pose and then sketch. And then in science when we were doing forces each lesson we got to do different things, like for the gravity lesson we got to make like little parachutes, and with friction we let little cars go down ramps.”

(Maisie)

Which Luke compares to the mundanity and helplessness of “just waiting”:

“I like it when I’m interacting in lessons… Instead of like, just waiting for someone just to keep talking and… sometimes it just gets a bit boring”

(Luke)

### Doing Well

Children’s accounts of what ‘doing well’ at school meant to them (research question 2) clustered across three superordinate GETs: getting it ‘all right’, people around you got it ‘all right’, and getting ‘a special sticker’.

#### Getting It all Right

All except three children equated ‘doing well’ to academic attainment, suggesting school afforded limited opportunities for children to feel competent. The three exceptions to this GET were children who spoke about other meaningful activities, all of which were outside school. Robert commented: “…what makes me feel well like, it’s not really school, but it’s tennis”. Robert’s delineation between school and tennis suggests school’s conception of doing well precluded non-academic activities. At school, children judged themselves against objective, impossibly high standards and were preoccupied with correctness:

“…getting everything, or nearly everything, right on a test that you’ve found really hard the lesson”

(Luke)

Kelsey: …if you get the work done, and it’s right and you get it done in time, it just makes you feel happy because you feel like you’ve accomplished something, so you’re proud.

Shannon: …you can get all this stuff done, but you won’t necessarily get it all right… then you might feel sad if you thought you got all of it right, and then when your books have been marked you find out that you’ve not got it all right.

The pride Kelsey derived from her “work” seemingly depended on it being “right” rather than the effort she put in; an experience shared by Shannon for whom getting “stuff done” is meaningless if it is not “all right”. Shannon’s sadness at thinking she “got all of it right”, then receiving her marked book only to “find out” she did not get it “all right” is palpable, her expectations impossibly high as she strives for “all” or nothing. Others were fixated with the number of correct answers, like John: “I need to work on my spellings and getting 10 out of 10. Or, like today I got seven”. Children’s’ concern about their number of “right” answers compared to others was also apparent:

Mayo: I was just very, shocked, how we only got six out of 10 when we’ve been practicing for four weeks? *[laughter]*

Fion: I got four out of ten!

Researcher: Why do you feel shocked?

Mayo: Because we’ve been practicing for four weeks.

Fion: And we… feel proud when we have 10 out of 10.

#### People Around You Got It all Right

Children described feelings of shame when their answers were incorrect, frequently comparing their ‘correctness’ to others’ expectations and expressing a desire for social validation.

Shannon: …you could feel really sad if, for instance, people around you had got it right but you hadn’t, then you could feel disappointed in yourself, or just… sad in general… if you’re not really doing anything fun or happy… it can stay on your mind for a long time.

Maisie: …but also… similar to what Shannon said, if you were doing work… and a lot of the time you’re used to getting everything right, so you do it right a lot… you might be doing questions that your teacher set you and you’re used to getting everything right, and your friends are used to you getting everything right. And then you do it, and they ask you ‘how many did you get right?’. Then it can be kind of embarrassing to say like, ‘Oh, I only got three or whatever.’

Shannon: Yeah, people ask around, ‘what did you get out of 10?’

Maisie: That kind of happened to me this morning, because we had a spelling test this morning, and usually I get 10 out of 10… you look through my book and every spelling test I’ve had was 10 out of 10. And then today I got eight which is only two off which isn’t really that bad because there are people who get like three or something. But it can still be a little bit embarrassing when people ask you ‘how many did you get?’, and they expect you to get 10 out of 10 because that’s what you’ve been getting constantly and then all sudden you like… drop down.

Kelsey: …just sort of adding on but, I think expectations can be a little bit… if a lot of people have high expectations of you and stuff, that can make you make you feel really like… overwhelmed, and pressured, and stressed and stuff….

Shannon: …it could be mood dropping if you know what I mean? …As in like so, you might be really happy or just neutral, and then if you’re embarrassed with that, or sad, then you could get sad.

Kelsey: That’s why people talk about a sinking feeling, that’s what it’s meant to describe. The ‘mood dropping’.

Whereas Kelsey and Maisie refer to the expectations of friends and “people” who “are used to you getting everything right”, Will described feeling “behind” on his “times tables” compared to the expected standard he is “supposed to” achieve:

“…in Year 6… you’re supposed to know all your times tables, and I’m a bit behind on my times tables so I really want to try to get learn all my times tables… if I’m going at fast speed, I’m like, I can do this, I’m nearly there… But if I’m going slow I can be “Ah I am never gonna do this” … I can feel either joyful and proud and excited… or… sad. And sometimes you can even feel embarrassed that everybody else can do something, and you can’t.”

(Will)

Will’s internalisation of the expected standard fuels his need to work at a “fast speed” to catch-up with “everybody else” who he perceives as being able to do something he “can’t”, expressing frustration at his “slow” speed and despondent inner voice telling him he is “never gonna do” it.

#### Getting ‘a Special Sticker’

Affirmation from teachers (“If a teacher’s given me good feedback”) was central to Eric feeling he had done well on his work, as Bob, John, and Robert also described:

Bob: …when the teacher says ‘you’ve done well’.

John: Oh, yeah. Like, one time my teacher said ‘brilliant work’ or something like that.

Researcher: How does that make you feel?

John: It makes me feel good.

Bob: Excited and proud.

John: Yeah, it makes me feel proud, happy, and positive about myself.

When asked whether verbal feedback was important, Robert elaborated that praise from teachers affirms his own feeling that he has “done well”, and that his success matters:

Robert: Because it’s it might be you’ve done well, but no one cares about it.

Bob: And when someone says you’ve done a good job, it feels like someone actually cares about you.

Both Robert and Bob needing “someone” to care is critical (see also ‘It feels like they don’t care’). Non-verbal feedback from teachers and getting recognition in their books felt equally meaningful to others. Children spoke of “special stickers” signifying worth and seemingly having a social currency as they remember clearly when *they* got a sticker, who *else* got one, and felt it important to tell others, as indeed Luke exclaims at the end of this exchange:

Asher: When I get a butterfly! …the sticker in your book, when you get a special sticker in your book!

Oman: For doing well

Luke: Yeah, every lesson, normally in maths, one person gets a special sticker… And whoever got that done really well

Asher: It was Kelsey who got it

Luke: I don’t remember…

Asher: In one lesson

Luke: I got it, I got it once!

## General Discussion

This phenomenological inquiry explored children’s school-specific experiences of hedonic and eudaimonic wellbeing. A phenomenological approach enabled children’s accounts to be unpacked in detail, attending to language and moments of co-construction. Providing closer examination of feelings and meanings at the root of ‘themes’ identified as important for children’s wellbeing in research is essential in challenging taken-for-granted, ‘adultcentric’ understandings of wellbeing (Ben-Arieh, 2005; Wright et al., 2022). Broadening analysis to include children’s eudaimonia is another important contribution of this study, with few studies eliciting children’s experiences of eudaimonia at school, despite eudaimonia’s educational relevance (Clarke, 2020).

### Feeling Good: Emotional Interdependence, Mistrust, and the Autonomy of Free Time

First, children described hedonic wellbeing as dependent on relatedness (*feeling cared for* and *showing care for* others), supporting Self-Determination Theory (SDT; Deci & Ryan, 2000) and research (Dex & Hollingworth, 2012). Children additionally articulated experiencing interdependence, deriving their own sense of wellbeing from the emotional states of those they spent most time with. Children described being highly attuned to the mood and behaviour of peers, naming this experience (of feeling good when their friends seemed happy) ‘the domino effect’.

Children’s sensitivity to peers’ behaviour was further articulated by their analogy ‘a pen can be stronger than a sword’, describing the lasting impact of cruel words spoken to them by others as more damaging than physical hurt, due to their lingering effect. Many struggled to forget cruel words spoken to them which ruminated internally for a long time despite not ‘wanting’ to ‘think about it’. Altogether, children’s lived experiences reinforce SDT, providing an additional nuanced understanding of how children experience wellbeing inter-individually (Waters et al., 2022). The implications of children’s emotional interdependence for schools? Children described the synchronicity of their wellbeing with their peers’ wellbeing, suggesting curricula focused on wellbeing literacy (Oades et al., 2021) could help equip children with tools to recognise, communicate and differentiate between their own and others’ experiences. Adults should also actively support children with conflict. For example, whole-school restorative approaches have proven efficacy in resolving conflict (Skinns et al., 2009; Thompson & Smith, 2018). Restorative justice engages entire school communities, enabling individuals harmed (adults or children) to communicate the impact of the harm to those responsible, have this acknowledged, resolved and mitigated in future.

Children described feeling *less good* at school when they felt uncared for or disregarded by adults, as found by Sabolova et al. (2020). What children’s accounts uniquely contribute is understanding of the overwhelming helplessness children experience in situations of conflict when peer groups splinter, and individuals become stuck ‘in between’ friends. Specifically, children described being unable to rely on adults for support with conflict resolution as exacerbating their helplessness. Children’s past attempts to gain adult support had left them feeling misunderstood, describing teachers as trivialising their predicaments and disregarding their feelings. In this study, asking adults for help was a case of ‘once bitten, twice shy’,^9^ with one unresponsive interaction undermining children’s trust in adults.

Others described adults’ insensitive responses to seemingly incidental playground moments which felt significant to children, leading them to believe adults were untrustworthy. One child’s mistrust in adults stemmed from a time he was physically ‘trampled on’ by another pupil with additional needs in the playground which was disregarded by staff. It is essential children felt cared for by, and trust in, adults at school, since safety is a foundational prerequisite for wellbeing (Maslow, 1943). Specifically, children feeling disregarded and their concerns being minimized by support staff highlight a need for greater staff reflexivity (Fook & Askeland, 2006; Thompson & Thompson, 2018). School leaders can prioritise professional development which enables deeper understanding of how adults’ interactions with children can impact children’s wellbeing and perceived levels of safety: both emotional and physical.

Finally, an experience negatively impacting children’s hedonia was their resentment of teachers using free time as a punitive sanction. Extending research suggesting sanctions erode children’s trust in adults (Montreuil et al., 2020) and that children regard their time as an important commodity (Yetunde et al., 2014) or form of agency (Fattore et al., 2009). Children described teachers unfairly punishing the whole class (‘cutting into’ or withholding break times) because of one pupil’s misbehaviour. Children’s frustration at their lack of autonomy over free time and how they choose to spend it offers another application of SDT in schools; providing a child perspective on how teachers can undermine autonomy (Ryan & Weinstein, 2009). To support children’s autonomy, school leadership should prioritise pupil voice (Anderson & Graham, 2016), establishing a school council for pupils’ to express concerns and have them escalated. Children’s rights-based approaches (Lundy, 2007) underline the need to consult children on all matters concerning them, including school priorities, how school is run, and any changes they want to be made. Indeed, the richness of the data collected herein using group IPA methodology demonstrates that children are capable of reflecting on their wellbeing experiences, when afforded the opportunity; discussing, differentiating, complementing and contradicting one another.

### Doing Well: Correctness, Expectations, and Shame

Children described internalising implicit messages signalled to them about their ‘welldoing’ at school and the detrimental impact of these messages on their hedonic wellbeing. Children’s language (of ‘correctness’, ‘failure’, and efficiency) conveyed a fixation with summative performance, cued by implicit and explicit forms of ‘feedback’ from teachers and peers. Children were attuned to extrinsic indicators of their ‘welldoing’ such as objective scores, peers’ responses to their scores, and signals from teachers including getting ‘a special sticker’, or verbal nods to performance. In contrast, children described feelings of shame when they did not ‘get it right’, staying on their ‘mind for a long time’. Children described being rewarded for performance rather than effort, internalised as ‘a lot of people’ having ‘high expectations’ of them. Consequently, children self-evaluated what their attainment meant about them as a person (‘everybody else can do something, and you can’t’).

Altogether, children’s experiences resonate with achievement motivation theories. Children’s preoccupation with ‘correctness’ and their ability relative to others could be attributable to a performance-oriented motivational climate favouring high ability and encouraging social comparison (Ames, 1992), undermining learning and motivation (Elliott & Dweck, 1988; Tuominen-Soini et al., 2008). How teachers provide feedback is one highly influential way of shaping children’s motivation and implicit beliefs about their abilities (Dweck, 1999). To tap into pupils’ intrinsic motivation to learn, teachers’ feedback should emphasise effort over ability, and accompany grades with guidance for how to improve (Ryan & Weinstein, 2009). Furthermore, children need to understand how to feel they are doing well *throughout the learning process*. To facilitate this, teachers can use Assessment for Learning practices (AfL) (Pyle et al., 2022) such as providing individualised feedback or one-to-one reflection.

Performance-oriented classrooms also breed social comparison (Elliot & Church, 1997), supported by children’s lived experiences herein. Children judged whether they had ‘done well’ dependent on peers’ performance, internalising shame when they did not achieve correctness (‘embarrassing to say, oh, I only got three’) and feeling ridiculed for scores (‘people ask around, what did you get out of 10?’). Children cited negative emotions (feeling ‘sad’ and ‘disappointed’ in themselves) during the public sharing of scores in the classroom, exposing when others ‘got it right’ and they did not, leaving them feeling humiliated. Unlike SDT (Deci & Ryan, 2000), for children in this study it was not *feeling* competent that mattered, but whether *others perceived them* as competent. Children’s accounts accord with Erikson’s psychosocial theory (1950), which highlights the central developmental task of late childhood: proving one’s competence versus inferiority to peers.

Altogether, children’s experiences highlight how classrooms can incubate negative emotions (Pianta et al., 2012) and the close relation between children’s hedonic and eudaimonic wellbeing at school. If children’s brains are preoccupied with fear and threat they are unlikely to engage in effective learning (Neville, 2013) as articulated by the earliest need theory (Maslow, 1943). To help children feel safe when learning, it is essential teachers provide private recognition of pupils’ efforts, rather than encouraging public showcases of performance, advocated by Epstein’s ‘recognition’ principle (TARGET; Epstein, 1989). Teachers can further use Epstein’s TARGET principles to cultivate positive motivational climates such as using varied and differentiated learning tasks.

### Limitations

Three limitations should be noted. The first being that findings were derived from children within just one school, due to the case study approach. Second, the sample was not sufficiently diverse compared to nationwide demographics. Specifically, children of certain non-White ethnicities, and children with a SEN or EHPC, could not be interviewed. Further, only one child interviewed was eligible for FSM. Further phenomenological inquiries are therefore required to understand the unique wellbeing experiences of these specific groups. This is especially imperative when it comes to children from particular non-White minority ethnic groups whose wellbeing experiences are likely to vary considerably from majority groups (see Stern et al., 2022) Third is the need to consider whether pre-interview engagement with the lead researcher may have influenced the experiences of school children chose to share. The wellbeing lessons, delivered as part of the main project (Clarke & Hoskin, 2022), may have primed children, leading them to discuss certain aspects of their experience of school over others. One activity involved pupils writing letters of encouragement to friends working towards learning goals at school, which may have led children to foreground achieving learning goals in interviews.

### Concluding Remarks

This study contributes to the literature in two main ways. First, it extends research suggesting children understand their wellbeing as both *feeling* good and *functioning* well (Vujčić et al., 2019), to illuminate how children’s experiences of the latter (eudaimonia) at school are instrumentalised as academic attainment. The constricted sources from which children felt able to derive feelings of competence (eudaimonia) in turn detrimentally impacted their happiness at school (hedonia). Children’s accounts of eudaimonia conveyed a fixation with summative academic performance, seemingly perceiving learning as a means to an end and lacking an understanding of the process or intrinsic worth of learning (Nicholls et al., 1989).

Second, it uncovered critical details of how children experience autonomy, relatedness, and care at school. Children’s autonomy was undermined when teachers did not listen to their concerns/seek their opinion about school, or honour free time. Relatedness to others was experienced as emotional interdependence, suggesting positive education (Kern & Wehmeyer, 2021) could help children understand their wellbeing as separate from, and related to, others’. Staff failed to facilitate the resolution of children’s conflicts and a culture in which acts of aggression were overlooked by adults resulted in children feeling distressed, unheard, and questioning the level of cared afforded to them whilst at school.

Throughout children’s’ experiences of doing well at school, there was a gnawing absence of curiosity or engagement in the wider purpose of learning. How, then, can teachers, accountable for children’s learning, make the learning process one that fosters children’s eudaimonic wellbeing? Eudaimonia is about feeling competent and having a sense of purpose. Teachers can engage children in the purpose and process of learning by connecting knowledge and skills to relatable, real-world experiences (Pianta et al., 2012). Further, children’s eudaimonia was undermined by an absence of formative feedback accompanying summative scores, meaning children lacked the skills or understanding required to improve their work. Teachers can help children feel competent by providing clear next steps for how to improve (Taras, 2005) and reward effort over ability.

The overarching concern of how heavily children’s experiences of ‘doing well’ at school focused on attainment, devoid of other pursuits, lingers. Notably, the three children mentioning non-academic activities took place *outside* school, suggesting they were not acknowledged as forms of ‘doing well’ *inside* school. Therefore, while children themselves understand that ‘doing well’ can be experienced broadly (Tuukkanen & Pekkarinen, 2022), adults at school may not recognise non-academic pursuits. The quality of an education is determined by the range of opportunities afforded for individuals to develop their ‘functionings’ (Sen, 1992). Accordingly, it is the duty of Government to embrace the variety of ways children experience competence by ensuring a broad and balanced curriculum and enabling school cultures that celebrate all forms of success, beyond academic achievement (Bonell et al., 2019).

## Supplementary Information

Below is the link to the electronic supplementary material.

ESM 1 (DOCX 23.7 KB)

## Data Availability

The datasets used and/or analyzed during the current study are available from the corresponding author on reasonable request.

## Footnotes

1 The term practitioners is used to refer to all adults working directly with children in the school context.

2 See supplementary materials.

3 Pseudonyms used throughout.

4 See Supplementary Materials.

5 Analysis of children’s responses to this section of the interview answered a third research question and is not presented in the present article.

6 Services included a range of local support such as specific charities and Child and Adolescent Mental Health Services as well as national services such as child helplines.

7 ‘Have you Filled a Bucket Today?’ by Carol McCloud.

8 Children’s allotted time to use the outdoor green space near their school is called ‘green time’.

9 ‘Once bitten twice shy is an English idiom used to mean that an individual who has failed/been hurt attempting to do something has become fearful or cautious about attempting to do the same thing again.
