# Supplementary material for: Children’s Lived Experiences of Wellbeing at School in England: a Phenomenological Inquiry
Source: Child Indic Res. 2023 Mar 3;16(3):963–96. doi: 10.1007/s12187-023-10016-2 (PMC9981449; doi:10.1007/s12187-023-10016-2)
Supplement: Supplementary file 1 — (DOCX 23.7 KB) [file 12187_2023_10016_MOESM1_ESM.docx]

**Children's lived experiences of wellbeing at school in England: a phenomenological inquiry**

**Supplementary Materials**

This document contains materials designed to supplement the main text. The materials include the following:

**Semi-structured interview schedule**

**Semi-structured interview schedule**

**Primary (Year 5 & 6) Pupils**

**Introduction**

- **Thank pupils for volunteering to participate, explain the purpose of the interview** (to explore in more detail the ideas they came up with in the wellbeing lessons, and their experiences of school more generally) and the research aim (to understand their experiences of feeling good and feeling they are doing well in school)
- **Explain the interview will last about 30 minutes**, but if they need to leave the room for a comfort break (they feel uncomfortable, or need the toilet), they can raise their hand at let me know any time
- **Explain the structure of the interview:** I’m going to ask you some questions, and then you should respond with any ideas you have in response. Ask pupils to try and let everyone have the chance to speak. Reiterate that just like in the wellbeing lessons, there are no right or wrong answers
- **Explain the rules of engagement:** just like in our wellbeing lessons, we had ground rules to make sure everyone felt comfortable. In our discussion now, we will also have rules. Clarify the ground rules: keep the conversation in the room*; non-judgemental approach; listen to others; right to pass.

*Establish basic safeguarding procedure for the interviews (provision of not offering complete confidentiality): if something you say in this interview makes me worry that you are unsafe, I will have to pass this on to someone in school who can ensure you receive the support you need.

Then quickly go through each ground rule in an interactive way. E.g. We will **listen to each other** (ask pupils what this means) and clarify based on their responses: this means not talking over one another, staying quiet when someone else is talking, but if you feel you have not had a chance to speak, you can raise your hand and I will make sure you have the chance to speak.

- Clarify that pupils do not have to share any feelings or ideas they do not feel comfortable sharing, and they should not share anything about themselves or others that is private.
- **Obtain pupil assent:** explain that now they know exactly what the interview will be, they are free to change their mind if they no longer want to take part. Explain nothing bad will happen to them if they decide they would rather not take part. Announce that we will take a minute to get set up to begin the interview; so if anyone has changed their mind they are free to leave the room *[and re-join the lesson/re-join playtime: ask a teacher to wait outside the room for the first 5 minutes to meet any pupil who does change their mind]*
- **Ask pupils to find their work from the wellbeing lessons** from the collection I have brought into the room (Good Advice Boxes and Letters to a friend). *[Wait 1-2 minutes in case any pupil has decided they no longer wish to take part.]*

**Main discussion**

**Ok, I think we are ready to begin then. First, I would like you to think back to the ‘Good Advice Box’ you created in our first lesson for an imaginary friend. Remember, they needed your advice to help them feel good and feel they are doing well in school. Explain that before we ‘officially’ begin, we are going to have a little warm-up exercise to remind us of some of the feelings we learnt about in our wellbeing lessons.**

**Starter activity using post-it notes on the table**

Q0.1) Can you name some different feelings you experience at school?

Q0.2) Which of these feelings might you experience when you **feel good** at school?

Q0.3) Which of these feelings might you experience when you feel **less good** at school?

**Well done! You have thought of lots of different feelings there. We can use them throughout our discussion now wherever you feel it might be helpful to refer to them. So, let’s think about these good feelings first.**

**Q1)** What type of things make you **feel good** in school?

1. **How** do you feel about…*[thing child mentioned]* /when you… *[thing child mentioned]*
2. **Why** do these things make you feel good?
3. **Can you tell me a bit more about…** *[thing child mentioned]*

*Prompts: what advice did you give your friend, to help them feel good?*

*Remember everything that makes up your experiences of school*

**Thank you, these are very interesting ideas. Now let’s think about the less good feelings you mentioned**

**Q2)** What are the things at school that make you feel **less good**?

1. **How** do you feel about… when you…
2. **Why** do these things make you feel less good?

*Prompt: what things can school pay more attention to, to make you feel good?*

**Thank you. Let’s move on now. Can you remember in our second lesson I asked you why we come to school? Let’s think about that again…**

Q0.4) Why do we come to school?

*[Eliciting pupils’ ideas about the purpose of school]*

**Q3)** What are the main things that make you feel **you are doing well** in school?

1. **When** do you feel you are doing well at school?
2. **Why** do these things make you feel you are doing well in school?
3. **How do you know** when you have done well at school?

*Prompt: In part one of our sessions together we imagined a classmate in your school who feels they are doing well; what were they doing exactly?*

**Q4)** Are there any things that make you feel you are **not doing well** at school?

1. **When** do you feel like you have not done well at school?
2. **What** is it about these things that make you feel you are not doing well at school?

**Thank you everyone, we are halfway through our discussion now. You are all coming up with some great ideas. Does anyone need to ask me any questions at this stage?**

*[pause for 1 minute]*

**Now let’s think back to the letter you wrote to your friend in our second lesson. Remember, your friend was working towards a learning goal. This is the type of goal – or ‘target’ that your teacher might set for your own learning journey. At times, your friend struggled, and work seemed hard. It was not always easy for them to achieve their goal. The letter you wrote was designed to give them words of encouragement and support.**

**Q5)** When you are working towards a learning goal at school, how does it make you feel?

1. **Why** do you feel that way?
2. **How** can we make sure that we still feel good at school, even when we are concentrating hard and putting a lot of work into our learning?

**Q6)** Is there anything about your experience of school that could be changed to make you feel good or feel you are doing well more often?

1. **How** would these changes make you feel good or feel you are doing well more often?

**This is my final question. I’d like to understand what you think about doing the kind of workshops we did together.**

**Q7)** Do you think having lessons about our feelings and experiences of school are important?

1. **Why** do you/don’t you think these types of lessons are important?

**General follow-up questions for unresponsive/shy pupils**

- That sounds interesting, can you tell me a little bit more about that?
- What makes you feel/think that?
- So *[Sarah]* thinks that *[restate Sarah’s point] -* does anyone have any other ideas? (for discussions where certain pupils talk more than others)

**Rounding off**

- **Thank pupils** for their time and ideas
- **Remind pupils who they can speak to in confidence** about their feelings if they ever feel they need to tell someone something that is worrying them or causing them to feel unhappy
- **Explain what will happen next**: I will go away and think about your ideas in relation to my research, but your ideas will not be linked to you personally. As a group, the ideas you gave me about how to make school a place that makes you feel good, will be fed back to school, to help make school a better place.
- **Ask pupils if they have any questions** for me about the research
